# Waterpipe Tobacco Smoking and Susceptibility to Cigarette Smoking Among Young Adults in the United States, 2012–2013

**DOI:** 10.5888/pcd13.150505

**Published:** 2016-02-18

**Authors:** Ramzi G. Salloum, M. Rifat Haider, Tracey E. Barnett, Yi Guo, Kayla R. Getz, James F. Thrasher, Wasim Maziak

**Affiliations:** Author Affiliations: M. Rifat Haider, Arnold School of Public Health, University of South Carolina, Columbia, South Carolina, and Jahangirnagar University, Dhaka, Bangladesh; Tracey E. Barnett, College of Public Health and Health Professions and College of Medicine, University of Florida, Gainesville, Florida; Yi Guo, Kayla R. Getz, College of Medicine, University of Florida, Gainesville, Florida; James F. Thrasher, Arnold School of Public Health, University of South Carolina, Columbia, South Carolina; Wasim Maziak, Stempel College of Public Health and Social Work, Florida International University, Miami, Florida, and Syrian Center for Tobacco Studies, Aleppo, Syria.

## Abstract

**Introduction:**

Waterpipe tobacco smoking, also known as hookah and shisha, has surged in popularity among young people in the United States. Waterpipe is also increasingly becoming the first tobacco product that young people try. Given the limited access to and limited portability of waterpipes, waterpipe smokers who become more nicotine dependent over time may be more likely to turn to cigarettes. This study examined the relationship between waterpipe tobacco smoking and susceptibility to cigarette smoking among young adults in the United States.

**Methods:**

Using data from the 2012–2013 National Adult Tobacco Survey, a nationally representative sample of US adults, we reported rates of current waterpipe smoking and susceptibility to cigarette smoking by demographic characteristics and by use of other tobacco products among survey participants aged 18 to 24 years. Multivariable logistic regression was used to examine the relationship between current waterpipe smoking and susceptibility to cigarette smoking, defined as the lack of a firm intention not to smoke soon or within the next year.

**Results:**

Of 2,528 young adults who had never established cigarette smoking, 15.7% (n = 398) reported being waterpipe smokers (every day or some days [n = 97; 3.8%] or rarely [n = 301; 11.9%]); 44.2% (176/398) of waterpipe smokers reported being susceptible to cigarette smoking. Those who smoked waterpipe rarely were 2.3 times as susceptible to cigarette smoking as those who were not current waterpipe smokers (OR = 2.3; 95% CI, 1.6–3.4).

**Conclusion:**

Current waterpipe smoking is associated with susceptibility to cigarette smoking among young adults in the United States. Longitudinal studies are needed to demonstrate causality between waterpipe smoking and initiation of cigarette smoking.

## Introduction

Waterpipe tobacco smoking, also known as hookah and shisha, has surged in popularity among young people in the United States (1,2) and worldwide (3). In the 2012–2013 National Adult Tobacco Survey (NATS), among all age groups who reported using tobacco, 18.2% of adults aged 18 to 24 reported waterpipe smoking (every day, some days, or rarely); this prevalence was the highest prevalence for any age category and for any tobacco product other than cigarettes (4). Waterpipe is also increasingly becoming the first tobacco product that young people try (5).

Young people may be attracted to waterpipe smoking because of its social allure (6) and the perception that it damages health less than cigarette smoking (7). Given the limited access to and limited portability of waterpipes, nicotine-dependent smokers may turn to cigarettes (8). Understanding the association between waterpipe smoking and susceptibility to cigarette smoking is the first step in exploring whether the appeal of waterpipes among young people could lead to initiation of cigarette smoking. Whereas waterpipe smoking is primarily an intermittent method of using tobacco (3), its disproportionate popularity among young people compared with older adults is particularly relevant because nearly all adults who become daily cigarette smokers initiate smoking by age 26 (9).

Susceptibility to smoking is defined as the lack of a firm decision against smoking, and it is a strong predictor of regular or established smoking among young people (10–12). Given the increasing prevalence of waterpipe smoking among young people in the United States, there is a need to understand whether it contributes to cigarette smoking susceptibility. A recent study observed subsequent cigarette smoking among waterpipe smokers at baseline (13); however, to our knowledge, this relationship has not been examined in a nationally representative US sample. Our objectives in this study were to describe the characteristics of young adults in the United States who are current waterpipe smokers and to determine whether current waterpipe smokers are more likely to report being susceptible to cigarette smoking than those who are not waterpipe smokers.

## Methods

Data were analyzed from the 2012–2013 NATS, a stratified, nationally representative telephone survey of 60,192 noninstitutionalized civilian US adults aged 18 years or older (4). The sampling design of NATS relies on independent samples drawn from households in the 50 US states and District of Columbia. The response rate for the 2012–2013 wave was 44.9% (landline frame, 47.2%; cellular frame, 36.3%). The analytic sample was limited to the 2,528 young adult (aged 18–24 years) respondents who had never established cigarette smoking behavior. Respondents were considered to be never-established smokers if they responded negatively to the question, “Have you smoked at least 100 cigarettes in your entire life?” and responded with “not at all” to the question, “Do you now smoke cigarettes every day, some days, or not at all?”

Current smokers of waterpipe tobacco were identified by using the following question: “The next question asks you about smoking tobacco in a hookah. A hookah is a type of water pipe. . . . Do you now smoke tobacco in a hookah every day, some days, rarely or not at all?” Participants who responded with either “every day” or “some days” were defined as current waterpipe smokers. Participants who selected “rarely” were defined as intermittent waterpipe smokers. Those who chose “not at all” were defined as nonsmokers of waterpipe.

The survey asked adults aged 18 to 29 the following 2 questions on susceptibility to future cigarette smoking: “Do you think you will smoke a cigarette soon?” and “Do you think you will smoke a cigarette in the next year?” Response options were “definitely yes,” “probably yes,” “probably not,” and “definitely not.” Those who responded with “definitely not” to both questions were considered not susceptible to cigarette smoking, whereas all other participants were considered susceptible. This classification is based on research showing that the smoking susceptibility measure predicts subsequent smoking behavior (10–12). We conducted a sensitivity analysis, which classified “definitely yes” and “probably yes” as being susceptible to smoking cigarettes and “probably not” and “definitely not” as being not susceptible to smoking. The NATS questionnaire did not include a question that is often used in assessing susceptibility to cigarette smoking (“If one of your best friends were to offer you a cigarette, would you smoke it?”), and thus our measure of susceptibility was constructed by using only 2 questions.

Associations between the current use of other tobacco products and susceptibility to cigarette smoking were also assessed. The tobacco product categories were the following: 1) cigars, cigarillos, and filtered little cigars; 2) smokeless tobacco, including chewing tobacco, dip, snuff, and snus; and 3) e-cigarettes. To establish current use, respondents were asked if they now used the product “every day,” “some days,” “rarely,” or “not at all.” Participants who responded with “every day,” “some days,” or “rarely” were considered current users of the tobacco product type. The association between harm perception and susceptibility to cigarette smoking was also assessed by using the following question: “How harmful do you think cigarette smoking is to a person’s health?” The response options were “not at all harmful,” “moderately harmful,” and “very harmful.” Finally, respondents were asked the following question to assess prior experimentation with cigarettes, “Have you ever tried cigarette smoking, even one or two puffs?” Those who responded affirmatively were deemed to have experimented with cigarettes at some point in their lifetime.

The following demographic characteristics of survey respondents were assessed: sex, age in years (18–21 or 22–24), race/ethnicity (non-Hispanic white, non-Hispanic black, non-Hispanic Asian, Hispanic, and non-Hispanic other), educational attainment (no high school diploma, high school graduate or general educational development [GED], some college or associate degree, bachelor’s degree or higher), and annual household income (<$20,000, $20,000–$49,999, $50,000–$99,999, ≥$100,000).

### Statistical analyses

Data were analyzed during August 2015 using Stata 14.0 (StataCorp LP) and weighted to adjust for the differential probability of selection and response. Landline data were weighted according to the selection probability of the telephone number, the number of adults in each household, and the number of telephone numbers per household. Cell telephones were assumed to be used exclusively by the respondent and were weighted only according to the selection probability of the telephone number. Final weights were adjusted for undercoverage by sex, age, race/ethnicity, educational attainment, and telephone type (ie, landline or cellular) (14). Demographic characteristics and rates of tobacco use were summarized by using weighted percentages and confidence intervals (CIs). Rao–Scott χ^2^ tests were used to detect significant differences between estimates that included corrections for survey design effects. Bivariate analyses were conducted to identify significant differences by current waterpipe smoking status. Multivariable logit regression was used to examine the relationship between respondent characteristics and susceptibility to cigarette smoking. Sensitivity analysis was used in the adjusted regression model to test the interaction of sex with current waterpipe smoking on susceptibility to cigarette smoking. This study was deemed exempt by the University of Florida institutional review board because it relied on publicly available data. 

## Results

Among respondents who had never established cigarette smoking (n = 2,528), 97 (3.8%) were current waterpipe smokers, and 301 (11.9%) were intermittent waterpipe smokers (Table 1). Among adults aged 18 to 24 years, most current waterpipe smokers were men (64.0%), aged 18 to 21 years (71.7%), had only a high school diploma or GED (67.7%), and had never tried cigarettes (74.5%). In this same age group, only 50.6% of intermittent waterpipe smokers were men. In addition, most intermittent waterpipe smokers were aged 18 to 21 years old (63.4%), non-Hispanic white (55.4%), had only a high school diploma or GED (70.6%), and had tried cigarettes (66.6%). 

**Table 1:** Characteristics of Sample of US Adults Aged 18 to 24 Years Who Never Established Cigarette Smoking (n = 2,528), National Adult Tobacco Survey, 2012–2013^a^

Characteristic	Never Established Cigarette Smoking^b^ (n = 2,528)	Waterpipe Smokers (n = 398)	
		Current (Every Day or Some Days) (n = 97)	Intermittent (Rarely) (n = 301)
**Sex**			
Female	53.2 (50.7–55.7)	36.0 (24.8–48.9)	49.4 (42.3–56.5)
Male	46.8 (44.3–49.3)	64.0 (51.1–75.3)	50.6 (43.5–57.7)
**Age, y**			
18–21	62.8 (60.6–65.0)	71.7 (60.1–81.0)	63.4 (56.9–69.5)
22–24	37.2 (35.0–39.4)	28.3 (19.0–39.9)	36.6 (33.0–42.3)
**Race/ethnicity**			
White, non-Hispanic	50.1 (47.8–52.4)	48.0 (36.2–60.0)	55.4 (48.7–61.9)
Black, non-Hispanic	11.3 (9.8–13.0)	4.3 (1.1–15.7)	5.8 (3.4–9.6)
Asian, non-Hispanic	5.5 (4.5–6.9)	7.1 (2.2–20.4)	4.3 (2.4–7.6)
Hispanic	20.7 (18.9–22.8)	20.6 (12.2–32.6)	22.2 (17.0–28.4)
Other, non-Hispanic	12.4 (11.0–13.9)	20.0 (12.7–30.1)	12.4 (8.9–17.0)
**Education**			
No high school diploma	13.2 (11.3–15.3)	17.3 (8.4–32.3)	6.9 (3.9–11.9)
High school graduate or GED	64.8 (62.5–67.2)	67.7 (54.0–78.9)	70.6 (64.1–76.2)
Some college or associate degree	9.0 (7.7–10.4)	8.3 (3.9–17.5)	5.3 (3.0–9.1)
Bachelor degree or higher	13.0 (11.8–14.4)	6.7 (3.0–14.2)	17.3 (13.3–22.3)
**Annual household income, $**			
<20,000	10.0 (8.8–11.5)	11.9 (6.2–21.6)	9.2 (5.9–13.9)
20,000–49,999	25.4 (23.4–27.5)	29.6 (20.2–41.1)	19.8 (15.1–25.6)
50,000–99,999	23.4 (21.5–25.5)	19.2 (11.3–30.6)	22.7 (17.2–29.4)
≥100,000	11.6 (10.1–13.2)	15.0 (7.6–27.7)	19.9 (15.1–25.7)
Unspecified	29.5 (27.4–31.8)	24.3 (15.1–36.7)	28.4 (22.8–34.8)
**Cigar/cigarillo smoking**			
Every day or some days	1.1 (0.7–1.7)	16.4 (8.8–28.6)	2.9 (1.4–5.8)
Rarely	3.7 (3.0–4.7)	26.4 (17.2–38.2)	13.3 (9.8–18.0)
Not at all	95.2 (94.1–96.0)	57.2 (44.8–68.7)	83.8 (78.8–87.8)
**Smokeless tobacco use^c^**			
Every day or some days	2.3 (1.8–3.1)	11.5 (5.9–21.0)	2.1 (0.9–4.9)
Rarely	0.7 (0.4–1.2)	5.3 (2.1–13.0)	1.0 (0.3–3.0)
Not at all	97.0 (96.1–97.6)	83.2 (72.9–90.1)	96.9 (94.0–98.5)
**E-cigarette use**			
Every day or some days	0.5 (0.2–1.1)	8.5 (3.8–18.1)	0.7 (0.1–3.6)
Rarely	1.4 (0.01–2.1)	27.4 (17.7–39.8)	6.7 (3.9–11.2)
Not at all	98.1 (97.2–98.7)	64.1 (51.6–74.9)	92.7 (88.0–95.6)
**Harm perception for cigarettes**			
Very harmful	92.8 (91.5–93.9)	64.2 (50.5–75.9)	90.8 (86.3–93.9)
Moderately harmful	6.3 (5.3–7.6)	30.5 (20.0–43.7)	9.2 (6.1–13.7)
Not at all harmful	0.9 (0.5–1.5)	5.3 (1.1–22.2)	0.0 (0.0–0.0)
**Experimentation with cigarettes**			
Former trier	36.9 (34.6–39.2)	25.5 (16.6–37.1)	66.6 (60.0–72.7)
Never smoker	63.1 (60.8–65.4)	74.5 (62.9–83.4)	33.4 (27.3–40.0)

Abbreviation: GED, general educational development.

a All values are weighted percentage (95% confidence interval).

b Respondents were considered to be never-established smokers if they responded negatively to the question, “Have you smoked at least 100 cigarettes in your entire life?” and responded with “not at all” to the question, “Do you now smoke cigarettes every day, some days, or not at all?”

c Includes chewing tobacco, dip, snuff, and snus.

Overall, among young adults who had never established cigarette smoking, 19.7% (95% CI, 17.9%–21.7%) of waterpipe smokers reported being susceptible to smoking cigarettes (Table 2). Almost half (48.0%; 95% CI, 29.4%–67.2%) of current waterpipe smokers and 42.8% (95% CI, 36.2%–49.7%) of intermittent waterpipe smokers reported being susceptible to cigarette smoking, whereas 16.2% (95% CI, 14.4%–18.3%) of those who were not current waterpipe smokers reported being susceptible. 

**Table 2:** Estimates and Adjusted Logistic Model of Factors Associated With Susceptibility to Cigarette Smoking Among US Adults Aged 18 to 24 Years Who Never Established Cigarette Smoking^a^ (n = 2,528), National Adult Tobacco Survey, 2012–2013

Characteristic	Susceptibility to Cigarette Smoking		
	Weighted % (95% CI)	*P* Value^b^	OR (95% CI)
**Overall**	19.7 (17.9–21.7)	—	—
**Waterpipe tobacco smoking**			
Every day or some days	48.0 (29.4–67.2)	<.001	1.9 (0.5–7.2)
Rarely	42.8 (36.2–49.7)		2.3 (1.6–3.4)^c^
Not at all	16.2 (14.4–18.3)		1 [Reference]
**Sex**			
Female	14.1 (11.9–16.8)	<.001	1 [Reference]
Male	25.6 (22.6–28.8)		1.6 (1.2–2.3)^c^
**Age, y**			
18–21	22.3 (19.8–25.1)	<.001	1.9 (1.3–2.7)^c^
22–24	15.3 (12.9–18.0)		1 [Reference]
**Race/ethnicity**			
White, non-Hispanic	18.7 (16.2–21.6)	.10	1 [Reference]
Black, non-Hispanic	17.0 (12.2–23.1)		0.9 (0.5–1.6)
Asian, non-Hispanic	13.7 (8.3–21.9)		1.3 (0.6–2.7)
Hispanic	23.4 (19.1–28.3)		1.3 (0.9– 1.9)
Other, non-Hispanic	22.6 (17.8–28.3)		1.4 (0.9–2.4)
**Education**			
No high school diploma	23.1 (17.2–30.4)	.16	1 [Reference]
High school graduate	20.0 (17.6–22.7)		0.8 (0.4–1.4)
Some college or associate degree	14.8 (10.2–20.9)		0.7 (0.3–1.4)
Bachelor degree or higher	16.8 (13.3–21.0)		0.9 (0.4–1.7)
**Annual household income, $**			
<20,000	20.8 (15.2–27.9)	.36	1 [Reference]
20,000–49,999	17.0 (13.8–20.8)		0.8 (0.5–1.3)
50,000–99,999	19.1 (15.4–23.4)		1.0 (0.6–1.6)
≥100,000	23.8 (18.4–30.3)		1.1 (0.6–1.9)
Unspecified	20.5 (17.1–24.3)		1.1 (0.7–1.9)
**Cigar/cigarillo smoking**			
Every day or some days	46.6 (26.2–68.1)	<.001	1.9 (0.6–6.2)
Rarely	44.4 (33.5–55.8)		1.4 (0.8–2.5)
Not at all	18.4 (16.6–20.4)		1 [Reference]
**Smokeless tobacco use^d^**			
Every day or some days	37.3 (24.9–51.6)	<.001	1.2 (0.6–2.4)
Rarely	42.5 (21.6–66.5)		1.4 (0.5–4.3)
Not at all	37.3 (24.9–21.1)		1 [Reference]
**E-cigarette use**			
Every day or some days	36.3 (9.8–74.9)	<.001	1.1 (0.1–21.6)
Rarely	60.6 (40.5–77.7)		2.0 (0.6–6.8)
Not at all	20.1 (18.0–22.3)		1 [Reference]
**Harm perception for cigarettes**			
Very harmful	18.4 (16.5–20.6)	<.001	6.5 (0.8–51.4)
Moderately harmful	36.7 (28.3–45.9)		11.2 (1.3–93.6)^c^
Not at all harmful	9.3 (2.0–33.4)		1 [Reference]
**Experimentation with cigarettes**			
Former trier	36.1 (32.4–40.0)	<.001	5.0 (3.6–6.8)^c^
Never smoker	10.1 (8.4–12.1)		1 [Reference]

Abbreviations: CI, confidence interval; OR, odds ratio.

a Respondents were considered to be never-established smokers if they responded negatively to the question, “Have you smoked at least 100 cigarettes in your entire life?” and responded with “not at all” to the question, “Do you now smoke cigarettes every day, some days, or not at all?”

b Calculated by adjusted logistic regression.

c Significantly different from reference group at *P* < .05.

d Includes chewing tobacco, dip, snuff, and snus.

In the adjusted logistic regression model, intermittent waterpipe smokers were 2.3 times as susceptible to cigarette smoking as those who were not current waterpipe smokers (OR = 2.3; 95% CI, 1.6–3.4). We observed no significant associations between current use of other tobacco products and susceptibility to cigarette smoking. In this model, men were more likely than women (OR = 1.6; 95% CI, 1.2–2.3) and young adults aged 18 to 21 years were more likely than those aged 22 to 24 years (OR = 1.9; 95% CI, 1.3–2.7) to report susceptibility to cigarette smoking. Young adults who perceived cigarettes as moderately harmful were significantly more likely (OR, 11.2; 95% CI, 1.2–93.6) than those who perceived them as not harmful to report susceptibility to cigarette smoking. Finally, respondents who had experimented with cigarettes at some point in their lifetime were more likely (OR, 5.0; 95% CI, 3.6–6.8) to report susceptibility to cigarette smoking than those who had never experimented with cigarettes. In the adjusted model, we found no significant differences in susceptibility to cigarette smoking by race/ethnicity, educational attainment, or annual household income.

The additional regression model testing the effect of sex on the relationship between current waterpipe smoking and susceptibility to cigarette smoking did not find a significant interaction effect between sex and current waterpipe smoking. In the sensitivity analysis, classifying only those who reported “definitely yes” or “probably yes” responses as susceptible to cigarette smoking resulted in a similar adjusted effect for current waterpipe smoking on susceptibility to cigarette smoking. Most control variables had similar effects in this model as they did in the original regression model.

## Discussion

Our study, using data from the 2012–2013 NATS, showed that among young adults who had never established cigarette smoking, 2 of 5 waterpipe smokers reported being susceptible to smoking cigarettes. Another study using the same data found that waterpipe smoking — primarily intermittent use — was the second-most prevalent (18.2%) tobacco use method (after cigarette smoking) among young adults in the United States (4). Our study is the first to examine the relationship between current waterpipe smoking and the susceptibility to cigarette smoking in a nationally representative sample of young adults in the United States.

Because longitudinal data sets that simultaneously track current waterpipe smoking and the initiation of cigarette smoking are lacking, an association between waterpipe smoking and the initiation of cigarette smoking is not clearly defined for young adult cohorts in the United States. Recent results from a longitudinal cohort study of adolescents in Jordan showed that waterpipe smoking at baseline led to initiation of cigarette smoking at follow-up (8). Another study that examined cigarette smoking susceptibility among waterpipe-smoking young people in the Middle East (15) reported that the odds of susceptibility to cigarette smoking were more than double for those who smoked waterpipe in the previous month than for those who did not. Until such data are available in the United States, the association between waterpipe smoking and the initiation of cigarette smoking will not be clearly defined. Nevertheless, our findings suggest that young adults who smoke waterpipe tobacco are at risk for cigarette smoking initiation because they did not express a firm intention to refrain from smoking cigarettes in the future.

Strengths of this study are the use of a large, nationally representative sample and the first-time assessment of the relationship between current waterpipe smoking and susceptibility to cigarette smoking. Limitations include the use of self-reported data, which are subject to recall bias. Second, because our data were cross-sectional, no inferences can be made about causality. Third, variables that may affect susceptibility to waterpipe tobacco smoking, such as whether parents, spouses, or others use tobacco, were not included in the NATS questionnaire. Fourth, although the measurement of susceptibility has predictive validity among adolescents (10–12), validity that we assume carries over to young adults, future research is required to test this assumption. 

To our knowledge, ours is the first US study to assess the relationship between current waterpipe smoking and susceptibility to cigarette smoking among young adults who had never established cigarette smoking. Although our study did not establish a cause-and-effect relationship between waterpipe smoking and cigarette smoking initiation, such a relationship is plausible, and longitudinal studies are needed to investigate its possibility. Given the high prevalence of waterpipe smoking among young people in the United States, it is important to assess both its direct and indirect consequences.
